# Dental Bibliography

**Published:** 1855-01

**Authors:** 


### ART. VI.—*Dental Bibliography.*

*The Anatomy, Physiology, and Pathology of the Human Teeth; with the most approved methods of treatment: including operations, and the method of making and setting Artificial Teeth. With thirty Plates.* By PAUL B. GODDARD, M. D., M. A. N. S., M. A. P. S., Demonstrator of Anatomy in Univ. of Penn., Lecturer on Anatomy, etc., etc. Aided in the practical part by JOSEPH E. PARKER, Dentist. New York: Samuel S. & Wm. Wood, 261 Pearl St. 1854.

This is a folio volume which we commend to the consideration of our dental readers. It gives a sufficiently full account of the development of teeth, preceded by a very interesting history of dental literature. The description of the microscopic anatomy of teeth, though not very full, is neat, concise, and intelligible, and moreover, beautifully illustrated.

The engravings are one of the strong features of the book, being very neatly drawn and handsomely lithographed. All parts of the volume, from the development of the tooth to the most delicate of those multiform instruments which dentists love to accumulate upon their tables, are faithfully depicted.

We are innocent of all practical dental knowledge or skill. When we attempt to pull a tooth it is at the risk of a fractured jaw to the unfortunate sufferer, and the operation is performed with a hand tremulous then, though unflinchingly steady in any other form of surgery.

Consequently we have long since abandoned the use of our limited dental armamentaria, and consign all toothaches to the tender mercies of the professional dentist.

The work before us is a very handsome volume, and will doubtless meet with a rapid sale.

For sale by PHINNEY & Co. H.

*A Dictionary of Medical Terminology, Dental Surgery, and the Collateral Sciences.* By CHAPIN A. HARRIS, M. D., D. D. S., Professor of Dental Surgery in the Baltimore College; M. A. M. S,, etc. Second edition, carefully revised and enlarged. Philadelphia: Lindsay & Blakiston. 1855.

The first large edition of this work having been exhausted, another has been issued to supply the continued demand.

We abstract from the preface the following paragraph which gives a correct idea of the alterations made from the original edition, and o the scope and character of the work:

"The present edition contains about eight thousand more words than the first. The introduction of these without very greatly increasing its size, which the author was anxious to avoid, rendered it necessary to re-write and compress the heavier and more elaborate articles into much narrower limits than were originally assigned to them, and to strike out the Bibliographical and Biographical departments altogether. The last was done the more willingly, as a work embracing these subjects, by a very able pen, has already been announced as in preparation. The character of the book in this respect being changed, a corresponding alteration of title became necessary. All the words, technicalities and other subjects belonging to Dental Surgery proper, have been retained, and all new terms, descriptions of subsequent discoveries and improvements in the art and science, have been carefully added. Numerous synonyms have also been introduced, and it is believed that no important word, in any of the specialties of Medicine, which has at all passed into general use, has been refused a place and a minute and careful definition in the present edition of the work."

For sale by Miller, Orton & Mulligan. H.

